# Jewish women’s headwear associated alopecia: a survey study

**DOI:** 10.1097/JW9.0000000000000212

**Published:** 2025-06-05

**Authors:** Ambika Nohria, Arianna Strome, Akshay Pulavarty, Nnaemeka Anyanwu, Jerry Shapiro, Fatima Bawany, Lina Alhanshali, Kristen Lo Sicco, Amy Bieber

**Affiliations:** a The Ronald O. Perelman Department of Dermatology, New York University Grossman School of Medicine, New York, New York; b Department of Dermatology, State University of New York (SUNY) Downstate Medical Center, Brooklyn, New York

**Keywords:** alopecia, hair loss, head coverings, religious headwear

**What is known about this subject in regard to women and their families?**

- Orthodox Jewish women may cover their hair for religious reasons, and case reports suggest an association between this practice and hair loss.
- Research on this association is limited despite the significance of hair to identity and beauty.
- Additionally, historically, a lack of culturally competent care has hindered patients’ access to and utilization of dermatology services.

**What is new from this article as messages for women and their families?**

- Over a third of respondents reported hair loss potentially linked to head coverings, with tighter hairstyles and certain attachment methods contributing to specific patterns.
- Women may reduce their risk by adopting looser styles, using less damaging attachments, or rotating attachment sites to protect their hair.
- This article highlights the importance of culturally competent care, emphasizing the need for providers to better understand and respect patients’ needs.
- This study identifies practical steps women can take while advocating for culturally sensitive, patient-centered care that empowers them to prioritize their hair health.

## Introduction

Many Orthodox Jewish women cover their natural hair after marriage, however, research on associated alopecia is limited.^1–5^ This study aims to better understand Jewish women’s experiences and explore the relationship between Jewish religious head coverings (HC) and alopecia, better informing culturally sensitive care.

## Methods

An online anonymous REDCap survey was circulated between July 14, 2024 and August 9, 2024 via text, email, and WhatsApp groups. Inclusion criteria included self-identifying as female, Jewish, and utilizing HC. Associations were evaluated with *χ*^2^ and Fisher’s exact test.

## Results

One hundred eighty-two women accessed the survey – 152 completed all required fields and met inclusion criteria. Most respondents were under age 45 (73.3%) and 76.6% reported “always” covering their hair, 62.3% for over 10 years. Respondents utilized various HC, including full wigs or sheitels (66.9%), half wigs or falls (48.1%), scarves or similar (72.7%), hats (37.0%), and others (7.1%) (Table 1).

**Table 1:** Patient demographics and experience of hair loss

Survey question	*n* (%)
Age (*n* = 154)	
18–25	4 (2.6)
26–29	16 (10.4)
30–35	49 (31.8)
36–39	19 (12.3)
40–45	25 (16.2)
46–49	11 (7.1)
50–55	11 (7.1)
56–59	2 (1.3)
60+	17 (11.0)
What type of head covering do you utilize? (*n* = 154)	
Full wig or sheitel^a^	103 (66.9)
Partial wig or fall^b^	74 (48.1)
Scarf, tichel^c^, or snood^d^	112 (72.7)
Hat or fascinator	57 (37.0)
Other	11 (7.1)
How often do you cover your hair? (*n* = 154)	
Less than once per week	9 (5.8)
Several hours per week	7 (4.6)
Several days per week	20 (13.0)
Always	118 (76.6)
How long have you been covering your hair on a regular basis? (*n* = 154)	
0–3 months	1 (0.7)
3–6 months	2 (1.3)
6 months–1 year	3 (2.0)
1–5 years	28 (18.2)
6–10 years	24 (15.6)
10+ years	96 (62.3)
Have you experienced hair loss associated with head covering use? (*n* = 152)	
Yes	52 (34.2)
No	100 (65.8)
What type of hair loss have you experienced? (*n* = 52)	
Frontal hairline, including temples	35 (67.3)
Side of head, including above and behind ears	3 (5.8)
Back of scalp	0 (0)
Diffuse thinning throughout entire scalp	22 (42.3)
At the attachment sites of the head covering	19 (36.5)
If you experience hair loss associated with head covering, how do you address this? (*n* = 52)	
Topical treatments	6 (11.5)
Oral treatments	2 (3.8)
Changing hairstyle underneath my head covering	10 (19.2)
Wearing head covering less often	10 (19.2)
Alternating location of clip/comb	7 (13.5)
Changing type of head covering	10 (19.2)
No treatment	26 (50.0)
Hair is important to identity? (*n* = 154)	
Strongly agree	63 (40.9)
Agree	54 (35.1)
Neutral	26 (16.9)
Disagree	6 (3.9)
Strongly disagree	5 (3.3)
Hair is important to beauty? (*n* = 152)	
Strongly agree	55 (36.2)
Agree	63 (41.5)
Neutral	26 (17.1)
Disagree	4 (2.6)
Strongly disagree	4 (2.6)
Would you be comfortable seeking care for hair loss? (*n* = 149)	
Yes	108 (72.5)
No	41 (27.5)
Who would you be comfortable seeking care from for hair loss? (*n* = 108)	
Primary care doctor	36 (33.3)
Dermatologist	64 (59.3)
Gynecologist	5 (4.6)
Religious authoritative figure	1 (0.9)
Other	2 (1.9)
Why would you not be comfortable seeking care for hair loss? (*n* = 40)	
Not enough head-covering-wearing female medical providers	4 (10.0)
Embarrassment	2 (5.0)
Lack of knowledge regarding religious head covering practices among medical providers	4 (10.0)
Lack of culturally competent care	7 (17.5)
Other	23 (57.5)
Hair loss is minimal	4 (10.0)
Hair loss is not important to me	9 (22.5)
I cover my hair anyways	4 (10.0)
Don’t want any unnatural treatments	2 (5.0)
I’d want to decide for myself whether to stop wearing a head covering	1 (2.5)
I’d rather wear my hair natural	1 (2.5)
Would not think there are solutions that meet my religious standards	1 (2.5)
Too taboo	1 (2.5)

a Sheitel: A full wig.

b Fall: A partial wig.

c Tichel: A scarf that is tied around the head covering all or part of the hair.

d Snood: A loose pouch- or hairnet-like covering, typically of opaque fabric.

HC perceived alopecia was endorsed by 34.2% of respondents. The most affected locations were frontal scalp/temples (67.3%), diffusely (42.3%), and HC attachment sites (36.5%) (Table 1). There was no significant association between the type of HC or duration of use with reported distributions of hair loss (Table 2).

**Table 2:** Associations between head coverings and hair loss

	Hair loss (*n* = 52)	No hair loss (*n* = 100)	*P*-value
Type of head covering	*n* (%)	*n* (%)	
Full wig/sheitel	39 (75.0)	62 (62.0)	.107
Partial wig/fall	29 (55.8)	43 (43.0)	.135
Scarf/tichel/snood	38 (73.1)	72 (72.0)	.888
Hat/fascinator	14 (26.9)	43 (43.0)	.052
Other	5 (9.6)	6 (6.0)	.512
Type of hairstyle			
Tight high bun or ponytail	8 (15.4)	6 (6.0)	.076
Loose high bun or ponytail	6 (11.5)	15 (15.0)	.557
Tight low bun or ponytail	22 (42.3)	37 (37.0)	.524
Loose low bun or ponytail	24 (46.2)	48 (48.0)	.829
Tight braid	1 (1.9)	4 (4.0)	.661
Loose braid	3 (5.8)	3 (3.0)	.412
Leave hair down	18 (34.6)	45 (45.0)	.218
Under cap	9 (17.3)	16 (16.0)	.837
Other	3 (5.8)	7 (7.0)	1.000
Method of head covering attachment			
Comb	11 (21.2)	22 (22.0)	.904
Headband	20 (38.5)	35 (35.0)	.674
Velvet	24 (46.2)	34 (34.0)	.143
Clips	34 (65.4)	50 (50.0)	.070
No attachment	36 (69.2)	71 (71.0)	.821
Other	1 (1.9)	8 (8.0)	.132
Duration of head covering practice			.679
0–3 months	0 (0.0)	1 (1.0)	
3–6 months	0 (0.0)	2 (2.0)	
6 months–1 year	2 (3.9)	1 (1.0)	
1–5 years	9 (17.3)	19 (19.0)	
6–10 years	9 (17.3)	15 (15.0)	
10+ years	32 (61.5)	62 (62.0)	

Women wear a variety of hairstyles under HCs, primarily tight low buns/ponytails (38.8%), loose low buns/ponytails (47.4%), and leaving hair down (41.4%). Compared to looser hairstyles, tighter hairstyles were associated with frontal hair loss (*P* = .039). The most common attachment methods were headbands (36.2%), velvet (38.2%), clips (55.3%), or no attachment method (70.4%). Clips had a greater association with hair loss (*P* = .07) than combs (*P* = .904), headbands (*P* = .674), velvet (*P* = .143), or no attachment method (*P* = .821) (Table 2).

Half of the women haven’t pursued treatment or changes when facing alopecia. Most women agreed or strongly agreed that hair is important to identity (76.0%) and beauty (77.7%), though 27.5% of women would not feel comfortable seeking care for alopecia, with 42.5% of those individuals endorsing concerns regarding cultural knowledge or sensitivity. (Table 1).

## Discussion

Our study highlights that perceived alopecia is a common issue amongst Jewish women who cover their hair, with over a third of those surveyed reporting hair loss associated with HCs. Additionally, most women consider hair important to their sense of identity and beauty, yet some may hesitate to seek treatment for alopecia due to concerns regarding culturally competent care. This underscores the need for improved provider knowledge and understanding to effectively treat Orthodox Jewish women.

Based on our results, healthcare providers may recommend looser hair styles under HCs, using alternatives to clips, moving sites of attachment methods, or opting for no attachment method. However, as demonstrated by the lack of significance in our findings, providers should keep a broad differential diagnosis beyond HCs as the cause of alopecia. These recommendations provide active solutions to alopecia while respecting patients’ religious practices.

Study limitations include sample size, response bias, reliance on self-reported data, and limited evaluation of alopecia severity. Further research is necessary to better evaluate alopecia within the Orthodox Jewish community to improve culturally competent care.

## Conflicts of interest

None.

## Funding

None.

## Study approval

NYU Langone Health IRB Approved (i24-00470).

## Author contributions

AN and AS: Contributed to study design, data collection, and manuscript preparation. AP and NA: Contributed to data analysis and manuscript review. JS, FB, LA, KLS, and AB: Contributed to study design and manuscript review.
